# The Relevance of Personal Characteristics in Allocating Health Care Resources—Controversial Preferences of Laypersons with Different Educational Backgrounds

**DOI:** 10.3390/ijerph9010223

**Published:** 2012-01-16

**Authors:** Jeannette Winkelhage, Adele Diederich

**Affiliations:** School of Humanities and Social Sciences, Jacobs University Bremen, Campus Ring 1, 28759 Bremen, Germany; Email: a.diederich@jacobs-university.de

**Keywords:** prioritizing, health behavior, conjoint analysis, distributive justice, distributive preferences, public attitudes, Germany, socialization theory

## Abstract

In all industrial countries publicly funded health care systems are confronted with budget constraints. Therefore, priority setting in resource allocation seems inevitable. This paper examines whether personal characteristics could be taken into consideration when allocating health services in Germany, and whether attitudes towards prioritizing health care vary among individuals with different levels of education. Using a conjoint analysis approach, hypothetical patients described in terms of ‘lifestyle’, ‘age’, ‘severity of illness’, ‘type of illness’, ‘improvement in health’, and ‘treatment costs’ were constructed, and the importance weights for these personal characteristics were elicited from 120 members of the general public. Participants were selected according to a sampling guide including educational background, age, chronic illness and gender. Results are reported for groups with different levels of education (low, middle, high) only. The findings show that the patients’ age is the most important criterion for the allocation of health care resources, followed by ‘severity of illness’ and ‘improvement in health’. Preferences vary among participants with different educational backgrounds, which refer to different attitudes towards distributive justice and might represent different socialization experiences.

## 1. Introduction

In all industrial countries publicly funded health care systems are confronted with an increasing demand for health care services on the one hand and limited financial resources on the other hand [1]. Priority setting in resource allocation seems inevitable [2]. While in Germany priority setting decisions are mostly based on the preferences of physicians [3,4], in other countries such as Sweden, Israel or Great Britain, prioritization guidelines have been developed by involving the general public [5]. The latter approach led to a greater acceptance of priority setting decisions and is furthermore a precondition for legitimacy and fairness [6]. 

Studies on the general public’s distributive preferences found that individuals take into account a number of justice principles for priority setting decisions in medicine including need [7,8], efficiency [9,10], and merit [9,11]. Prioritization according to need or ‘rule of rescue’ [12] implies that health care services should be allocated to those in the greatest medical need, defining need as severity of illness [1]. That is, those who are the most severely ill should be given priority in treatment. An allocation according to efficiency aims at maximizing health care benefits from a given budget [1]. Priority is given to those with the greatest capacity to benefit (e.g. health gain after treatment) and to those whose treatment is less expensive [13,14]. Priority is furthermore given to younger over older people assuming that the younger live longer and consequently benefit more from treatment [15]. The merit principle points to the people’s lifestyle and therewith to the question of whether individuals have increased their risk of morbidity with unhealthy behaviors, such as smoking or excessive alcohol consumption. Because they are not responsible for their illness, priority is given to those with a healthy lifestyle who deserve a treatment more than those with an unhealthy lifestyle [9].

Studies show that severity of illness [8,16,17,18] and therapeutic benefit [16,17,19,20] are strongly supported by the public as criteria for priority setting in medicine. For instance, when asked to prioritize casualties in a triage situation, 89% of participants in a national opinion survey would prioritize causalities with severe pain over those with mild pain, and 58% would prioritize casualties with high chances of survival over those with low chances of survival [16]. 

However, the empirical evidence on whether the public accept personal characteristics, such as lifestyle or self-infliction of disease [7,20,21], and age [7,22,23,24] for priority setting decisions is less clear. The same is true for treatment costs [14,25,26]. The results are inconsistent and seem to be influenced by study design, the framing of questions, and nationality. For instance, Diederich and Schreier [21] found a majority of participants in a national opinion survey supporting higher charges for medical services for drug users (76%), extreme athletes (74%), for people who consume much alcohol (71%), smokers (68%), and for people who sunbathe or visit a solarium (65%); whereas Shmueli [20] reports from another national opinion survey where only 3% of respondents considered health behavior as important in allocating medical services. Age as a criterion to prioritize health care was supported in a focus group study done by Cookson and Dolan [7] who found that interviewees gave priority to the younger patients; whereas Mossialos and King [24] found a majority of respondents in a cross national opinion survey rejecting age based priority setting. Participants in a conjoint based study done by Schwappach and Straßmann [25] prioritized treatments with costs below average; but 81% of respondents in an opinion survey done by Nord *et al.* [14] thought that priority should not depend on treatment costs.

Studies on priority setting in medicine examining differences in preferences within the general public showed that personal characteristics such as educational achievement have an impact on public opinion [11,18,20,24]. For instance, Ryynänen [18] found that higher education was associated with a higher endorsement of age, severity of disease and prognosis of disease as criteria for priority setting. Mossialos and King [24] showed that survey participants with higher education had a greater preference for allocations according to treatment outcome than participants with lower education, whereas no differences between participants with different educational backgrounds regarding age based priority setting have been found. Oddsson [27] did not find any differences between the public’s preferences that could be attributed to personal characteristics. Findings concerning the acceptance of various priority setting criteria by different population groups are therefore still inconclusive. But knowledge about such differences is crucial since they raise difficulties when developing criteria acceptable to the whole population [24].

Furthermore, little empirical evidence exists on the relative importance people of the general public attach to different priority setting criteria [13,28,29]. In most studies respondents have been asked to consider a maximum of two criteria at the same time [11,14,18,27] with fewer studies investigating at least three criteria simultaneously [30,31,32]. However, most studies that have investigated more than two criteria at the same time have either not estimated weights for those criteria [31] or have addressed priority setting decisions for very specific contexts only (e.g., Rodriguez-Miguez *et al.* [32] consider a waiting list for cataract extraction); see Green and Gerard [10] for an exception. Reviews of the literature around distributive preferences for health care therefore concluded that research is needed on the relative importance of competing criteria for priority setting [13,28,29]. 

In this paper, we investigate the relative importance of several criteria for priority setting in health care, including those that are controversially discussed by the general public, such as lifestyle, age, and treatment costs. We use a conjoint analysis (CA) [33]. This technique has been widely used for market research since the 1970s, and gained prominence within health research in recent years [34]. The major advantage of this method is that individuals consider several attributes or criteria simultaneously, and are forced to make trade-offs between them to come to a decision; thus the relative importance of each attribute can be generated as well as the part-worth utility for each attribute level [33].

The objective of the present study was to investigate criteria and underlying principles of distributive justice used by a sample of the German population to prioritize health services. In Germany only few attempts have been made to involve the general public in the process of priority setting in medicine [16]. Here, a study is reported where 120 laypersons were asked to rank order hypothetical patients who differed in age, lifestyle, type and severity of illness, improvement in health and treatment costs by priority of treatment. None of the participants had a medical profession but were familiar with the health care system as patients. We put a special focus on differences in the preferences of individuals with different educational backgrounds; we compare the attitudes of persons with lower education (without vocational training and final-secondary school examinations), middle education (completed vocational training or final-secondary school examinations), and higher education (university (of applied science) degree). A few studies in Germany have investigated the relative importance of different priority setting criteria, however, with students from the medical and economics faculties [25,35], or have not examined differences in the preferences of individuals with different educational backgrounds [22]. To our knowledge, no study exists which examines the values people with different levels of education attach to a set of competing priority setting criteria for prioritizing health care services in Germany. 

## 2. The Determination of Attitudes Towards Distributive Justice in Health Care: Hypotheses

We assume that attitudes towards distributive justice in medicine may be influenced by the person’s education. To explain how such differences in preferences may arise we make use of socialization theory, which was proven to explain differences between individuals with different levels of education in other contexts of distributive justice, as for instance, in the context of welfare state attitudes [36].

According to socialization theory individuals internalize specific norms and values which determine their attitudes, beliefs and skills, and by which they learn to perform as a member of their society [37,38]. Socialization is a complex and interactive process that continues throughout the life-course, involving purposeful learning processes as well as mechanisms like observation and imitation [39,40]. Depending on the individual’s phase of life it is moderated by certain agents, for instance, the family, school and working place [38]. Socialization is not equal for all members of society but differs with social variables like educational achievement [38]. Specific socialization processes lead to different attitudes, beliefs, and skills. Differences in attitudes towards priority setting in medicine can therefore, be explained in terms of different socialization processes. For instance, Kohn *et al.* [41] found that children whose parents are more advantageously located in the social structure of their society are more likely to learn values and skills such as intellectual flexibility or self-direction than those whose parents are less advantageously located in the social structure of their society. These skills and values learned by higher status children are necessary to acquire knowledge, to gain educational success and to reproduce the social status of their parents [42]. Educational success leads according to Andreß and Heien [36] to “individual success ideologies” among the better educated; that is, individuals who are more successful in the educational system will be more of the opinion that individual achievement is profitable and should be rewarded than those who are less successful in the educational system. We therefore hypothesize that individuals with higher education are more likely to support the merit principle than individuals with lower education. Moreover, one could assume that individuals with higher education believe that not only personal achievement but achievement in general should be rewarded. We therefore hypothesize that individuals with higher education are more likely to support the efficiency principle than individuals with lower education.

In the present study, principles of distributive justice were linked to prioritization criteria in the following way: The merit principle was represented by the attribute healthy lifestyle. People who support the merit principle have a greater preference for patients with a healthy lifestyle over those with an unhealthy lifestyle. The efficiency principle was represented by the attributes age, improvement in health, and treatment costs. People who support the efficiency principle have a greater preference for younger over older patients, they prioritize treatments with a large health improvement, and those with low costs. 

Table 1 summarizes the attributes—the prioritization criteria representing the principles of distributive justice—and the expected direction of increasing preference for patients with the respective attribute level. 

**Table 1:** Attributes representing the principles of distributive justice.

Principle	Attribute	Preference
**Merit**	Lifestyle	Healthy ≻ unhealthy
**Efficiency**	Health improvement	High ≻ low
	Treatment costs	Small ≻ large
	Age	Young ≻ old

Note: ≻ = is preferred to

## 3. Methods

To investigate the distributive preferences of laypersons with different levels of education and to test whether people with higher education have a greater preference than people with lower education for patients with a healthy lifestyle, younger patients, patients with a large health gain after treatment, and patients who involve low costs, a conjoint analysis (CA) technique was used. CA is designed to reveal individuals’ preferences for multi-attribute alternatives. Based on preference judgments of choice alternatives as a whole, CA allows to estimate simultaneously the so-called part-worth utilities for the different levels of each attribute to establish their impact on the overall utility of the alternative [43]. For the present study a questionnaire was developed which consisted of a CA ranking exercise. The alternatives were hypothetical patients requiring a medical treatment, and the attributes were criteria describing these patients that were considered as relevant in determining their priority of treatment. Respondents were asked to rank order the hypothetical patients according to priority of treatment. CA was developed in the following steps: identifying attributes and levels, presentation of scenarios, selection of respondents, data analysis.

### 3.1. Attributes and Levels

The attributes and levels for the hypothetical patients were obtained from an explorative interview study with 32 laypersons of the German population on prioritizing health care. Participants were selected on the basis of extreme case sampling to contrast low with high educational background (without vocational training and final secondary-school examinations *vs.* with university (of applied science) degree), young with old age (18–44 years *vs.* >64), chronically ill people with people without chronic diseases, and men with women. An interview guide was used to explore priority setting criteria and underlying principles of justice used by laypersons to prioritize medical services or groups of persons. Interviews were subjected to content analysis, coding frequencies were determined, and Fisher’s Exact Tests were estimated to reveal differences in the interviewees’ use of the allocation criteria. For the inclusion in the CA, criteria were selected that were mentioned by at least 30% of interviewees, and that reveal significant differences between participants or that were controversially discussed. The literature suggests not to choose more than six attributes to avoid an information overload [43]. Moreover, the number of levels across attributes should be balanced to prevent a number-of-levels effect, *i.e.*, people tend to give more importance to attributes defined by more levels [44]. 

The following attributes and levels were included in the CA as dependent variables.

*Age*. This attribute refers to the patient’s age at the time of illness. The literature suggests using specific numbers rather than ranges to limit the scope of interpretation for the participants [44]. Age was therefore assigned the concrete levels of 16, 37 and 68 years, representing adolescents, people of working age, and retired persons, respectively.

*Healthy lifestyle*. This attribute was assigned the levels ‘yes’ and ‘no’, describing the patient’s lifestyle as either healthy or unhealthy. A patient with a healthy lifestyle was characterized as non-smoker, with at most moderate alcohol consumption, healthy eating habits, and as taking sufficient exercise. A patient with an unhealthy lifestyle is not conforming to one or more of these characteristics.

*Type of illness*. This attribute was assigned the levels ‘chronic’ and ‘acute’, describing the patient’s illness. A patient with a chronic disease was described as taking regularly pharmaceuticals or as being at least quarterly in need of medical treatment; whereas a patient with an acute disease requires medical treatment without being chronically ill.

*Severity of disease*. The patient’s severity of disease indicates the health state of patients before the treatment and was classified as ‘light’ or ‘severe’.

*Improvement in health*. This attribute refers to the therapeutic benefit of a treatment and the associated level of health gain for the patient. It was assigned the levels ‘small’, ‘middle’ and ‘large’.

*Treatment costs*. The costs for the patient’s treatment were classified as ‘low’, ‘medium’ and ‘high’.

Attributes, attribute descriptions, and assigned levels are summarized in Table 2.

**Table 2:** Attributes and levels included in the Conjoint Analysis.

Attribute	Description	Levels
**Age (AGE)**	Patient’s age at the time of illness.	16 years
		37 years
		68 years
**Healthy lifestyle (LIFESTY)**	Whether a patient can be characterized as non-smoker, with at most moderate alcohol consumption, healthy eating habits, and as taking sufficient exercise versus not meeting one or more of these conditions.	Yes
		No
**Type of illness (ILLN)**	Patient’s type of illness.	Chronic
		Acute
**Severity of illness (SEV)**	Severity of patient’s disease before treatment.	Light
		Severe
**Improvement in health (IMPR)**	Patient’s health gain after treatment.	Small
		Middle
		Large
**Treatment costs (COST)**	Costs for the patient’s treatment.	Low
		Medium
		High

### 3.2. Presentation of Scenarios

The attributes and levels gave rise to 216 (2^3^ × 3^3^) possible scenarios, *i.e.*, hypothetical patients. The literature suggests that the number of scenarios should not exceed 30 for being manageable for the respondent [43]. The SPSS (17.0) ORTHOPLAN procedure was used to produce a fractional factorial design where the number of 216 possible scenarios was reduced to 16. The underlying orthogonal design ensures that the interattribute correlation is zero, and allows estimating main effects, *i.e.*, the utility of each attribute level, holding all other attributes constant [45].

A ranking exercise was used to elicit preferences for the hypothetical patient scenarios. We considered this data collection method more suitable than rating or paired comparisons because: first, ranked data is considered to be more reliable than rating data, because it is easier for the participant to decide which patient should get a higher priority than to decide on the magnitude of this priority [43]; second, a ranking exercise is more realistic, since top priority is given to only one patient [46]; third, a ranking exercise is more efficient than paired comparisons, because it requires less scenarios to be compared or, assuming the scenarios are the same, a smaller sample size [44]. That is, 16 scenarios would require 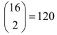 paired comparisons which might have overburdened the participants.

The ranking exercise was introduced by explaining the hypothetical patient scenarios, the attributes and their respective levels. Each scenario was printed on separate cards and the respondents’ task was to rank order all 16 patient scenarios according to priority of treatment. To ease the task, the respondents were instructed to sort all cards into two or three piles according to lower and higher priority, rank order the cards within each pile, merge the piles and then check the complete order for consistency. Afterwards, participants numbered the 16 cards consecutively from one to 16, where a one indicated the highest priority for being treated first and a 16 the lowest priority for being treated first. Table 3 shows one out of 16 hypothetical patient cards used for the ranking task.

**Table 3:** Hypothetical patient card for the ranking task.

Patient 14	Rank ___
Age	37 years
Healthy lifestyle	No
Type of illness	Acute
Severity of illness	Light illness
Improvement in health	Middle improvement
Treatment costs	Low

Questions about the respondents’ personal data appeared at the end of the questionnaire. Respondents were asked about their sex, age, lifestyle (whether they smoke, how much alcohol they consume, how much exercise they have), height and weight to determine the Body Mass Index, whether they have a chronic illness, whether they have a statutory or private health insurance, and about the highest level of education they have achieved. 

### 3.3. Selection of Respondents

The CA was conducted between June and October 2009. Participants were selected from the general population of Bremen as a purposive sample, *i.e.*, sample selection took place corresponding to the information richness of cases for the research question [47]. To carry out different perspectives on prioritization a heterogeneous sampling was performed [47]; respondents were selected according to a sampling guide that combined the criteria *education*, *age*, *sex* and *health status*. *Education* was assigned the levels ‘without vocational training and final-secondary school examinations’, representing individuals with lower education, ‘completed vocational training or final-secondary school examinations’, representing individuals with middle education, and ‘university (of applied science) degree’, representing individuals with higher education [48]. *Age* was assigned three levels: ‘18–44 years’, ‘45–64 years’, and ‘65 years and above’. *Sex* was classified as ‘male’ and ‘female’, and *health status* was classified as ‘chronically ill’ and ‘not chronically ill’. 

Subjects were primarily contacted by advertisements in local newspapers. In addition, subjects were directly contacted in randomly selected public utility institutions such as helpdesks for unemployed persons, since there was little feedback from persons with lower education to the newspaper advertisements. An incentive of 8 Euros and travel expenses, if necessary, were offered. Those who responded to the advertisements were invited to Jacobs University Bremen; all others were interviewed in those institutions where the recruitment took place. The first author asked each participant for written informed consent; then, she distributed the questionnaire, answered questions, if necessary, and collected the questionnaire after completion. To guarantee anonymity, informed consent was separated from the questionnaire. 

### 3.4. Data Analysis

Data analysis was carried out with SPSS (17.0). The CA was performed by the procedure CONJOINT. The model was defined as DISCRETE since all attribute levels were categorical and no assumption was made about the relationship between the attributes and the ranks. The SEQUENCE subcommand was included to account for the ranking procedure, starting with the most preferred patient card and ending with the least preferred patient card. CA assumes a compensatory utility model and the overall utility, *U*, of a patient profile, *k*, is equal to the sum of the part-worth utilities, *u_j_*, across its attribute levels *m* [44], *i.e.,*


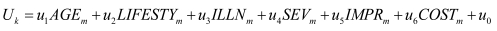


where *u*_0_ is a constant and interpreted as basis utility, estimated by the mean rank across all ranks. The part-worth utility scores, *u_jm_* refer to the desirability of each attribute level. In the present analysis no interactions between attributes were assumed. From estimated parameters the relative importance of each attribute, *i.e.*, the relative impact of an attribute upon the overall utility, was calculated by dividing its utility range by the sum of the ranges for all attributes. For details and procedure see [43,44,45,49] and electronic supplementary information (Text S1).

For subgroup analysis part-worth utilities and the relative importance of attributes were calculated for participants with lower, middle and higher education separately. ANOVA and Kruskall-Wallis H-test were utilized to reveal differences in preferences between these groups of individuals; the first was applied to normally distributed dependent variables and the latter to variables that did not fulfill the condition of normal distribution. If significant differences arose, either Bonferroni test, Dunnett T test, or Mann-Whitney U test, respectively, were applied as follow-up test to investigate the influence of different levels of education [50]. 

To test the hypotheses of whether participants with higher education have a greater preference than participants with lower education for patients with a healthy lifestyle, younger patients, patients with a high level of health gain after treatment, and patients who involve low costs, the respective part-worth utilities of both groups were compared using Dunnett T test for normally distributed dependent variables, and the one sided Mann-Whitney U test otherwise. 

### 3.5. Validity

The validity of the results was tested at aggregated levels (all groups together) using different approaches: Internal validity was assessed by the correlation (Pearson’s r) between the estimated and the input parameters of the dependent variable. The results from the regression analysis were also used to test the theoretical validity, *i.e.*, to what extent results are in line with previously formulated expectations. That is, we assumed that individuals would prioritize a healthy lifestyle, severe illness, large improvement in health, and low treatment costs. Predictive validity was tested by the correlation (Spearman coefficient) between the predicted rank order of the patient scenarios generated by the model and the average rank order given by participants. 

## 4. Results

### 4.1. Sample

A total of 121 individuals completed the questionnaire. Data from one participant were excluded because equal ranks were given to different patient cards. From the remaining 120 respondents 28 (23%) belonged to a group with lower education, 57 (48%) belonged to a group with middle education, and 35 (29%) belonged to a group with higher education. 61 respondents (51%) were female, and 59 were male; 55 (46%) were chronically ill, and 65 were not. Mean and standard deviation of their age were 51, and 17 years, respectively. Table 4 reports the sample characteristics.

**Table 4:** Descriptive characteristics of respondents.

Characteristics	n	%
**Sex**		
Male	59	49
Female	61	51
**Age**		
18–44	40	33
45–64	51	43
>64	29	24
**Education**		
Lower education	28	23
Middle education	57	48
Higher education	35	29
**Health status**		
Chronically ill	65	54
Not chronically ill	55	46

Note: N = 120.

### 4.2. Estimated Model

Table 5 reports the groups’ overall estimated part-worth utilities for each attribute level and the attribute’s relative importance (information on the range of part-worth utilities are found in Table S1, see electronic supplementary information). With the exception of the part-worth utilities estimated for treatment costs, all other parameters were significant at the 1% level, and the 5% level, respectively. The results provide support for the theoretical validity of the model, since respondents prioritized a healthy lifestyle, severe illness, large improvement in health, and low treatment costs. Furthermore, the respondents prioritized younger patients and those with an acute illness, keeping all the remaining attribute levels the same. Age had the highest relative importance value, closely followed by severity of disease and improvement in health. The model showed a good internal validity (Pearson’s r = 0.993) and predictive power (Spearman’s rho = 0.898).

**Table 5:** Groups’ overall estimated part-worth utilities and relative importance of each attribute.

Attribute	Level	Part-worth utilities (Standard error)	t-statistic (p*)	Relative importance
**Age**	16 years	0.75 (0.14)	4.101 (0.000)	20.9%
	37 years	−0.25 (0.17)	−1.986 (0.049)	
	68 years	−0.50 (0.17)	−2.541 (0.012)	
**Healthy lifestyle**	Yes	0.98 (0.11)	6.789 (0.000)	15.3%
	No	−0.98 (0.11)	−6.789 (0.000)	
**Type of illness**	Chronic	−0.48 (0.11)	−3.925 (0.000)	11.2%
	Acute	0.48 (0.11)	3.925 (0.000)	
**Severity of illness**	Light	−1.46 (0.11)	−9.479 (0.000)	19.6%
	Severe	1,46 (0.11)	9.479 (0.000)	
**Improvement in health**	Small	−1.26 (0.14)	−8.912 (0.000)	19.5%
	Middle	0.37 (0.17)	3.912 (0.000)	
	Large	0.89 (0.17)	5.510 (0.000)	
**Treatment costs**	Low	0.15 (0.14)	1.330 (0.186)	13.5%
	Medium	0.05 (0.14)	0.490 (0.625)	
	High	−0.20 (0.14)	−1.418 (0.159)	
**Constant**		8.59 (0.12)		

Note: N = 120; * p ≤ 0.05.

CA allows determining the utility for all attribute level combinations. The magnitude of the part-worth utility value indicates the importance of an attribute level relative to the overall utility of a choice alternative, *i.e.*, the higher the value the more it contributes to the overall sum. For the current study, a 16 year-old patient with a healthy lifestyle who suffers from a severe and acute illness, whose treatment is inexpensive and would largely improve his/her health has the highest priority for being treated first. The overall utility for this patient card is

U = 8.59 + 0.75 + 0.98 + 0.48 + 1.46 + 0.89 + 0.15 = 13.3

The lowest priority for a treatment receives a 68 year old patient with an unhealthy lifestyle who has a light and chronic illness, whose treatment is expensive and would lead to a small improvement of health, *i.e.*, 

U = 8.59 + (−0.50) + (−0.98) + (−0.48) + (−1.46) + (−1.26) + (−0.20) = 3.71

The constant is interpreted as basis utility and the attribute levels add to it or reduce it. The relative importance reflects the range of the part-worth utility within a given attribute related to the range of part-worth utilities across all attributes. Note that the values in Table 5 are based on the ranking of all participants. Relative importance does not mean that the attribute and its levels contribute greatly to the overall utility. It indicates, however, that the preference structure may change substantially by varying the attribute levels. 

### 4.3. Subgroup Analysis

**Figure 1:**
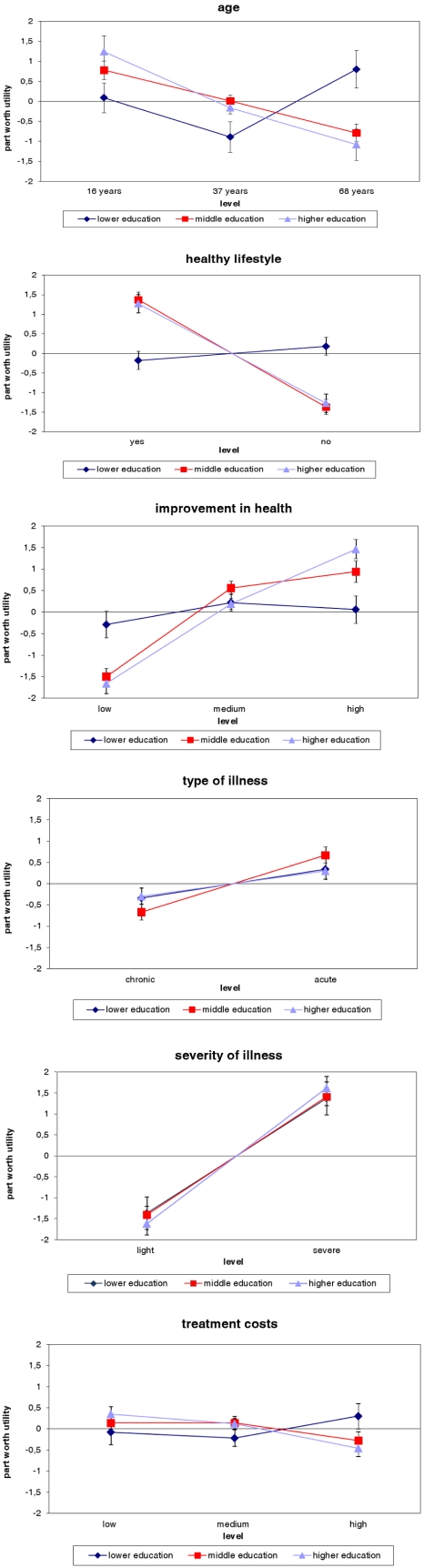
Part-worth utilities estimated for participants with lower, middle and higher education separately.

Part-worth utilities estimated for participants with lower, middle, and higher education separately revealed differences in the preferences between these groups (Figure 1; statistical details are found in Table S2, see electronic supplementary information): Participants with lower education preferred younger and older patients over middle aged, showing non-monotonicity in preference as a function of age, whereas participants with middle and higher education preferred younger over older patients, showing monotonicity in preference. Participants with a higher education preferred patients with an unhealthy lifestyle less than participants with a lower education did; but the former group preferred patients with a healthy lifestyle more than the latter group did. Furthermore, improvement in health was valued higher by participants with higher education than by those with lower education. No statistically significant differences were found regarding treatment costs, type of illness, and severity of illness.

Pairwise comparisons of part-worth utilities revealed differences between participants with lower and middle education (Table 6) and between participants with lower and higher education (Table 7). No differences in the preferences were found between participants with middle and higher education (results not shown). Differences between part-worth utilities estimated for participants with lower and middle education are as follows: Participants with lower education weighted old age, unhealthy lifestyle, and small improvement in health higher than participants with middle education, whereas the latter had a higher preference for a healthy lifestyle (Table 6).

**Table 6:** Differences between part-worth utilities estimated for participants with lower and middle education.

Attribute	Level	Part-worth utility (Standard error)		Mean difference (p*)
		Lower education n = 28	Middle education n = 57	
**Age**	37 years	−0.89 (0.38)	0.01 (0.15)	−0.895 (0.102) ^1^
	68 years	0.80 (0.47)	−0.79 (0.22)	−1.586 (0.011) ^1^
**Healthy lifestyle**	Yes	−0.18 (0.23)	1.37 (0.19)	−4.310 (0.000) ^2^
	No	0.18 (0.23)	−1.37 (0.19)	−4,310 (0.000) ^2^
**Improvement in health**	Small	−0.29 (0.31)	−1.66 (0.23)	1.371 (0.001) ^3^
	Large	0.06 (0.32)	1.46 (0.22)	0.878 (0.083) ^3^

Note: ^1^ Dunnett T test; ^2^ Mann-Whitney U test; ^3^ Bonferroni Test; * p ≤ 0.017.

**Table 7:** Hypotheses testing: Differences between part-worth utilities estimated for participants with lower and higher education.

Attribute	Level	Part-worth utility (Standard error)		Mean difference (p*)
		Lower education n = 28	Higher education n = 35	
**Age**	16 years	0.09 (0.37)	1.24 (0.40)	−2.064 (0.020) ^2^
	68 years	0.80 (0.47)	−1.08 (0.39)	−1,881 (0.000) ^1^
**Healthy lifestyle**	Yes	−0.18 (0.23)	1.27 (0.27)	−3.362 (0.000) ^2^
	No	0.18 (0.23)	−1.27 (0.27)	−3.362 (0.000) ^2^
**Improvement in health**	Small	−0.29 (0.31)	−1.66 (0.23)	−1.214 (0.000) ^1^
	Large	0.06 (0.32)	1.46 (0.22)	1.402 (0.001) ^1^
**Treatment costs**	Low	−0.08 (0.30)	0.35 (0.18)	0.425 (0.143) ^1^
	High	0.30 (0.30)	−0.46 (0.20)	−0.765 (0.040) ^1^

Note: ^1^ one sided Dunnett T test; ^2^ one sided Mann-Whitney U test; * p ≤ 0.017.

Differences between participants with lower and higher education provide support for the hypotheses (Table 7): Participants with lower education weighted unhealthy lifestyle and small improvement in health higher than participants with higher education, whereas the latter had a higher preference for a healthy lifestyle and large improvement in health. Participants with lower education weighted an old age higher than participants with higher education, but unlike than expected, no statistically significant differences were found for a young age. Contrary to our expectations, both groups valued similarly low and high treatment costs.

Note that the part-worth utilities within the criteria age, healthy life style and improvement in health varied less for the group with lower education than for the remaining two groups. That means for this group different levels of these attributes contributed little to the overall utility to distinguish between patient profiles. 

## 5. Discussion and Conclusions

‘Lifestyle’, ‘age’, ‘severity of illness’, ‘type of illness’, ‘improvement in health’, and ‘treatment costs’ are possible criteria that could be taken into account when setting priorities in health care. However, there is little information on how to trade off competing values. This is especially the case for Germany, where a public debate on priority setting in medicine is still lacking. This study used a conjoint analysis approach to investigate the relative importance of criteria for prioritizing health care in Germany taking into account the perspective of laypersons. It revealed differences in the preferences of individuals with different levels of education; hypotheses deduced from socialization theory were partly supported by the data. 

We hypothesized that socialization processes affect preferences for the merit and the efficiency principle of justice, and suggested a greater support of these principles by individuals with higher education. We therefore predicted that individuals with higher education have a greater preference than individuals with lower education for patients with a healthy lifestyle (this criterion was representing the merit hypothesis), younger patients, patients with a large health gain after treatment, and patients who involve low costs (these criteria were representing the efficiency hypothesis). This prediction only holds for healthy lifestyle and large health gain after treatment. The latter is in line with the findings reported by Nord [11].

Differences in preferences between individuals with middle and lower education revealed that the first group is more in favor of the merit principle. Attitudes towards the efficiency principle varied only slightly. No differences in preferences between participants with middle and higher education were found. The findings may reflect similar socialization experiences of the latter groups. 

To sum up, participants with higher and middle education were more in favor of the merit principle, which was represented by healthy lifestyle, than participants with lower education. Our findings with respect to the efficiency principle are less clear; *i.e.*, the effect of education on attitudes towards an efficiency-based allocation of health care resources is in question. This suggests that we have to consider other variables, such as age or sex, which may help to reveal differences in the general public’s preferences for priority setting in the German health system. 

An allocation of health care resources according to lifestyle is highly problematic. First, it is difficult to determine whether a certain condition was caused by unhealthy behavior rather than genetic, societal or environmental factors [51]. Moreover, the adoption of healthy lifestyles is influenced by socioeconomic factors, such as education [52]. Lower priority for patients with an unhealthy lifestyle could increase health inequalities in a society [53]. According to Leist [54], personal responsibility requires equal opportunities for healthiness; he therefore suggests prioritizing individuals who are in poor health due to social or genetic inequalities. In conclusion, health inequalities should be taken into account when setting priorities in health care. 

The *relative* importance values of the criteria investigated in this study were similar among participants with different levels of education. Although the specific part-worth utilities are different in each group the relative magnitude is about the same across groups, e.g., high for age, low for cost. That is, there is a general agreement of the importance of prioritization criteria across participants with different educational backgrounds. As this is the first CA study that includes education as explanatory variable further investigation is necessary.

We found that age, severity of illness, and improvement in health had the highest importance values. The importance of severity of illness and improvement in health for the allocation of health care resources is in common with previous studies reported above. However, the extent to which respondents favored age as priority setting criterion is surprising and the reasons can only be hypothesized. Studies that have investigated the relative importance of different prioritization criteria have found only limited support for age as priority setting criterion [22,25,35], while respondents of this survey found age to be the most important criterion. Wirsik *et al.* [49] describe similar results from a conjoint-based study conducted in Germany. Both findings might represent the current health policy debate in Germany where the impact of demographic ageing on publicly funded health care systems is an often-stated topic. Since insurance contributions are linked to wages, an increasing old-age dependency ratio causes drop in revenue, and thus financial problems [55]. Note that our study and the Wirsik *et al.* study [49] included ranking tasks while Schwappach [25,35] and Diederich *et al.* [22] presented discrete choice tasks. It seems implausible that the format should be responsible for the different results. However, we cannot exclude it entirely. Further research is needed here. 

The concrete levels of age (16, 37 and 68 years) also might have caused a systematic bias since all other criteria are rather abstract, with levels such as ‘light’ and ‘severe’. For instance, in their conjoint-based study, Wirsik *et al.* [49] found that respondents’ preferences for the allocation of health care resources varied depending on whether hypothetical patients in a choice set were described in general terms (e.g., having a severe disease, an unhealthy lifestyle, and a high occupational status) or in concrete terms (e.g., having lung cancer, being a smoker, and business leader). They divided participants into two groups; the groups were asked to rank order either the abstract or the concrete patient cards. A statistical test revealed a significant difference between the two groups regarding the distribution of the relative importance of the attributes. However, the authors did not examine whether a certain abstract attribute got a higher weight than its concrete proxy, and vice versa. Further research is needed to get reliable information on this topic. 

We rule out that the importance of age is a result of a response-ordering effect, according to which the great importance of this criterion would be due to its first position on the patient card [56]. Otherwise, ‘severity of illness’ and ‘improvement in health’ should have had lower importance values than ‘healthy lifestyle’ and ‘type of illness’, since they appeared towards the bottom of the patient cards [49]. Farrar and Ryan [56] who explicitly examined the impact of the ordering of attributes on the importance respondents attached to them did not find any evidence of a response-ordering effect.

There are some reasons against age-based priority setting. First of all, it is in conflict with the European rule of equality, which ban ageism by law [57]. Chronological age is furthermore a rather arbitrary criterion since biological age may vary strongly [58]. Here, criteria such as ‘severity of illness’ or ‘therapeutic benefit’ might be more appropriate. However, supporters of aged based priority setting consider age as a rather fair criterion since it affects all individuals equally because everybody is aging [57]. The controversial discussion about age based priority setting did not correspond to our findings, which might stem from the lacking public debate about prioritizing health care services in Germany. This underlines the necessity to foster public involvement in this country.

A methodological weakness of the present study is that no interactions among attributes have been analyzed. For instance, Rodríguez and Pinto [59] found that age interacts with health gain. However, to provide the respondents of our study with a manageable number of patient scenarios, a main effects design had to be used. Further research is needed to investigate interactions.

Participants of our study selected themselves which may have caused a self-selection bias, *i.e.*, it is possible that people who have a substantial knowledge or a personal interest in health care allocation were more willing to participate in the study than those who have not. However, a sampling guide ensured that different personal criteria were included in the sample so that the problem of sampling bias was alleviated. Further studies should attempt to substantiate our results in a larger, representative sample of the German general population.

In conclusion, this study is, to our knowledge, the first that examines the relative importance persons with different educational backgrounds attach to a set of competing criteria for prioritizing health care services in Germany. The revealed differences in the respondents’ preferences point out the necessity not to involve only people with higher education in the medical decision making process, as this could tighten health inequalities. Further studies with larger representative samples are necessary to confirm our findings.

## Supplementary Files

Supplementary File 1: PDF-Document (PDF, 239 KB)
