# Supplementary material for: The Relevance of Personal Characteristics in Allocating Health Care Resources—Controversial Preferences of Laypersons with Different Educational Backgrounds
Source: Int J Environ Res Public Health. 2012 Jan 16;9(1):223–43. doi: 10.3390/ijerph9010223 (PMC3315072; doi:10.3390/ijerph9010223)
Supplement: Supplementary File 1: — PDF-Document (PDF, 239 KB) [file ijerph-09-00223-s001.pdf]

Int. J. Environ. Res. Public Health **2012**, *9*, 223-243; doi:10.3390/ijerph9010223

OPEN ACCESS

International Journal of
Environmental Research and Public Health
ISSN 1660-4601
www.mdpi.com/journal/ijerph

Supplementary Information

The Relevance of Personal Characteristics in Allocating Health Care Resources—Controversial Preferences of Laypersons with Different Educational Backgrounds

Jeannette Winkelhage * and Adele Diederich

School of Humanities and Social Sciences, Jacobs University Bremen, Campus Ring 1, 28759 Bremen, Germany; E-Mail: a.diederich@jacobs-university.de

* Author to whom correspondence should be addressed; E-Mail: j.winkelhage@jacobs-university.de; Tel.: +49-421-200-3435; Fax: +49-421-200-3303.

Received: 03 August 2011; in revised form: 12 December 2011 / Accepted: 11 January 2012 / Published: 16 January 2012

Abstract: In all industrial countries publicly funded health care systems are confronted with budget constraints. Therefore, priority setting in resource allocation seems inevitable. This paper examines whether personal characteristics could be taken into consideration when allocating health services in Germany, and whether attitudes towards prioritizing health care vary among individuals with different levels of education. Using a conjoint analysis approach, hypothetical patients described in terms of 'lifestyle', 'age', 'severity of illness', 'type of illness', 'improvement in health', and 'treatment costs' were constructed, and the importance weights for theses personal characteristics were elicited from 120 members of the general public. Participants were selected according to a sampling guide including educational background, age, chronic illness and gender. Results are reported for groups with different levels of education (low, middle, high) only. The findings show that the patients' age is the most important criterion for the allocation of health care resources, followed by 'severity of illness' and 'improvement in health'. Preferences vary among participants with different educational backgrounds, which refer to different attitudes towards distributive justice and might represent different socialization experiences.

Keywords: prioritizing; health behavior; conjoint analysis; distributive justice; distributive preferences; public attitudes; Germany; socialization theory

---

Text S1: Conjoint Analysis and its Procedure

Conjoint analysis (CA) has its origin in the theory of conjoint measurement [1], which, based on specific axioms, proves that an ordering structure, for example some preference order, can be mapped into a numerical scale that exceeds an ordering scale; this means it can be mapped into an interval scale. While measurement-theory oriented research mainly focuses on the conditions for the existence of these scales and on the composition rule of the attribute scale values (e.g., additive, multiplicative) [2,3], users, *i.e.*, mainly researchers in marketing, are more interested in scaling aspects, especially for calculating specific numerical scale values [4,5]. In recent years, conjoint analysis and discrete choice theory, a generalization of CA [6], has also been applied in health research [7].

Conjoint analysis consists of several components that need to be specified prior to the study: preference function; data collection method; experimental design; presentation of choice alternatives; measurement of dependent variable; estimation of model parameters [4].

Here the preference function was described by a linear model without interaction; the data collection method included full profiles, *i.e.*, hypothetical patients were described by all attributes; the design was orthogonal, *i.e.*, a fractional factorial design excluding interaction terms between attribute levels; the choice alternatives (hypothetical patients) were cards, *i.e.*, patient profiles were printed on cards and shown to the participant; the participant rank ordered the cards according to his/her preferences of treating the hypothetical patients; parameters were estimated by ordinary least square methods.

Conjoint analysis is an individual analysis, *i.e.*, it analyses the preference structure of a single person; this is shown in the following derivations. To determine the preference structure of a specific population the results of all members of that group are aggregated, or, alternatively, the rankings of all participants are interpreted as repeated measurements.

The Model

The model in its general form is defined as

$$U_k = u_0 + \sum_{j=1}^{J} \sum_{m=1}^{M_j} u_{jm} \cdot x_{jmk} + \varepsilon_k$$

where U_k is the overall utility value of the choice alternative *k*, *i.e.*, the hypothetical patient described on the *k*-card.

u_0 is a constant reflecting the average rank of all given rank values. It is interpreted as basis utility.

u_{jm} is the part-worth utility value for attribute $j, j = 1 \ldots J$ with level *m*. *J* is the total number of attributes, here $J = 6$.

x_{jmk} is an indicator variable and takes on the value 1 if choice alternative *k* has attribute *j* with level *m*, and zero otherwise.

ε_k is a residual error which is an identically and independently normally distributed random variable with $N(0.s^2)$.

Conjoint analysis seeks to determine the part-worth utility values u_{jm} such that the overall utility value, U_k, reflects the empirical rank value, R_k. This can be achieved by calculating the empirical

average rank value for each single attribute level, $\overline{R_{jm}}$, and subtract the estimated basis utility value, $\hat{u}_0 = \frac{1}{K}\sum_{k=1}^{K} R_k$ from them. That is, $\hat{u}_{jm} = \overline{R_{jm}} - u_0$. In the present study with 16 ranks, $\hat{u}_0 = 8.5$.

Then the part-worth utility values are determined by minimizing the sum of squared deviation between the empirical and estimated utility values, *i.e.*,

$$\min \sum_{k=1}^{K} (R_k - U_k)^2$$

Relative importance

The magnitude of the part-worth utility value indicates the importance of an attribute level relative to the overall utility of a choice alternative, *i.e.*, the higher the value the more it contributes to the overall sum. However, it does not reveal the relative importance of an attribute. That is, if an attribute has similar values for all its levels, regardless of whether the values are low or high, any of these attribute levels contributes about the same to the overall utility. Therefore, little change in preference can be expected. More important for a preference change is the *range* of levels, *i.e.*, the difference between the highest and the lowest part-worth utility. This is captured in a relative importance measure for each attribute, ω_j. The range of levels of an attribute is related to the sum of level ranges of all attributes, *i.e.*,

$$\omega_j - \frac{\max_m(u_{jm}) - \min_m(u_{jm})}{\sum_{j=1}^{J}[\max_m(u_{jm}) - \min_m(u_{jm})]}$$

Utility values of groups

(a) Aggregation

To aggregate data across participants, the part-worth utilities of each single person are normalized to guarantee that they have the same origin and the same scale unit.

To set the origin for each attribute, the level with the lowest utility value, $u_j^{\min}$, is set to zero and the remaining levels are adjusted accordingly, *i.e.*,

$$u_{jm}{*} = u_{jm} - u_j^{\min}$$

The normalized part-worth utility is expressed in terms of the adjusted part-worth utility relative to the sum of the largest adjusted part-worth utilities of all attributes, *i.e.*,

$$\hat{u}_{jm} = \frac{u_{jm}{*}}{\sum_{j=1}^{J} \max_j(u_{jm}{*})}$$

(b) Repeated measurement

The rankings of all participants are treated as repeated measurements of the design. The equations are according to the model presented in **The Model** except that the number of choice alternatives is now $N \cdot K{*}$, with N the number of participants. All rankings are taken simultaneously for estimating the part-worth utilities. This procedure preserves the information contained in the variability and was applied for the current study.

Note that the estimates obtained from this procedure are the basis for determining the relative importance values within each group. A wide range of part-worth utilities of given attribute levels, *i.e.*, a large variability within the population increases the relative importance index of that attribute.

For a practical application with SPSS codes see e.g., Backhaus *et al.* [8].

Table S1. Groups' overall estimated part-worth utilities and their ranges.

Attribute	Level	Part-worth utilities (Standard error)	Minimum	Maximum
**Age**	16 years	0.75 (0.14)	−5,33	5,33
	37 years	−0.25 (0.17)	−6,00	3,67
	68 years	−0.50 (0.17)	−5,13	6,00
**Healthy lifestyle**	Yes	0.98 (0.11)	−3,63	4,00
	No	−0.98 (0.11)	−4,00	3,63
**Type of illness**	Chronic	−0.48 (0.11)	−4,00	4,00
	Acute	0.48 (0.11)	−4,00	4,00
**Severity of illness**	Light	−1.46 (0.11)	−4,00	4,00
	Severe	1,46 (0.11)	−4,00	4,00
**Improvement in health**	Small	−1.26 (0.14)	−5,33	2,67
	Middle	0.37 (0.17)	−2,04	4,25
	Large	0.89 (0.17)	−3,33	5,21
**Treatment costs**	Low	0.15 (0.14)	−5,33	2,67
	Medium	0.05 (0.14)	−2,21	2,58
	High	−0.20 (0.14)	−3,67	4,83
**Constant**		8.59 (0.12)	6,92	10,42

Note: N = 120.

Table S2. Differences between part-worth utilities estimated for participants with lower, middle, and higher education.

Attribute	Levels	Part-worth utility (Standard error)			F^1	χ^2	p *
		Lower education n = 28	Middle education n = 57	Higher education n = 35			
**Age**	16 years	0.09 (0.37)	0.78 (0.23)	1.24 (0.40)			0.077
	37 years	−0.89 (0.38)	0.01 (0.15)	−0.16 (0.15)	4.28	5.14	0.017
	68 years	0.80 (0.47)	−0.79 (0.22)	−1.08 (0.39)	7.53		0.001
**Healthy lifestyle**	Yes	−0.18 (0.23)	1.37 (0.19)	1.27 (0.27)		19.43	0.000
	No	0.18 (0.23)	−1.37 (0.19)	−1.27 (0.27)		19.43	0.000
**Type of illness**	Chronic	−0.34 (0.25)	−0.67 (0.19)	−0.30 (0.19)	0.99		0.374
	Acute	0.34 (0.25)	0.67 (0.19)	0.30 (0.19)	0.99		0.374
**Severity of illness**	Light	−1.37 (0.39)	−1.41 (0.21)	−1.62 (0.27)	0.22		0.803
	Severe	1.37 (0.39)	1.41 (0.21)	1.62 (0.27)	0.22		0.803
**Improvement in health**	Small	−0.29 (0.31)	−1.50 (0.19)	−1.66 (0.23)	8.24		0.000
	Middle	0.22 (0.20)	0.56 (0.16)	0.19 (0.12)	1.73		0.183
	Large	0.06 (0.32)	0.94 (0.25)	1.46 (0.22)	5.30		0.006
**Treatment costs**	Low	−0.08 (0.30)	0.14 (0.14)	0.35 (0.18)	0.92		0.400
	Medium	−0.22 (0.20)	0.14 (0.15)	0.12 (0.12)	1.29		0.280
	High	0.30 (0.30)	−0.28 (0.21)	−0.46 (0.20)	2.19		0.117

Note: [1] ANOVA; [2] Kruskall-Wallis H-test; * $p \leq 0.05$.

References

1. Luce, D.R.; Tukey, J.W. Simultaneous conjoint measurement: A new type of fundamental measurement. *J. Math. Psychol.* **1964**, *1*, 1-27.
2. Krantz, D.; Luce, R.D.; Suppes, P.; Tversky, A. *Foundations of Measurement*; Academic Press: New York, NY, USA, 1971; Volume 1, pp. 1-624.
3. Falmagne, J.C. Random conjoint measurement and loudness summation. *Psychol. Rev.* **1976**, *81*, 65-79.
4. Green, P.E.; Srinivasan, V. Conjoint analysis in consumer research: issues and outlook. *J. Consum. Res.* **1978**, *5*, 103-123.
5. Green, P.E.; Srinivasan, V. Conjoint-analysis in marketing—New developments with implications for research and practice. *J. Mark.* **1990**, *54*, 3-19.
6. Louviere, J.; Hensher, D.A.; Swait, J.D. *Stated Choice Methods. Analysis and Application*; Cambridge University Press: Cambridge, UK, 2000; pp. 1-418.
7. Ryan, M.; Gerard, K.; Amaya-Amaya, M. Using Discrete Choice Experiemts to Value Health and Health Care. In *The Economics of Non-Market Goods and Recources*; Bateman, I.J., Ed.; Springer Verlag: Dordrecht, The Netherlands, 2008; volume 11, pp. 1-276.
8. Backhaus, K.; Erichson, B.; Plinke, W.; Weiber, R. *Multivariate Analysemethoden: Eine Anwendungsorientierte Einführung*, 12th ed.; Springer: Heidelberg, Germany, 2008.

© 2012 by the authors; licensee MDPI, Basel, Switzerland. This article is an open access article distributed under the terms and conditions of the Creative Commons Attribution license (http://creativecommons.org/licenses/by/3.0/).
